# 289. Post COVID Syndrome Cohort Characterization

**DOI:** 10.1093/ofid/ofab466.491

**Published:** 2021-12-04

**Authors:** Bhoomija Chatwani, Shelby Flaherty, Sharon Liu, Marc Theberge, Mark Zeller, Kristian Anderson, Matt Boisen, Luis Branco, Robert Garry, Arnaud Drouin, Dahlene Fusco

**Affiliations:** 1 Tulane School of Public Health and Tropical Medicine, New Orleans, Louisiana; 2 Tulane School of Medicine, New Orleans, Louisiana; 3 The Scripps Research Institute, San Diago, California; 4 Zalgen Labs, Germantown, Maryland

## Abstract

**Background:**

Post COVID Syndrome (PCS) is significant morbidity following COVID-19. This study aims to identify biomarkers that predict PCS in a Gulf Coast cohort known for poor health outcomes.

**Methods:**

Since March 2020 the study Collection of Serum and Secretions for SARS CoV-2 Countermeasure Development (aka ClinSeqSer) has been enrolling subjects with confirmed acute COVID-19, with initial visit at 1 month and follow up every three months from symptom onset. At follow-up, subjects complete symptom questionnaire, physical examination, nasopharyngeal swab/saliva collection, blood draw. Subjects with >= one symptom new since COVID are PCS, remainder are Non-PCS experienced at initial one month visit and six months or longer. Univariate and bivariate analysis was carried out to study significant associations of currently available dataset (N=60).

Figure 1. Post-COVID Symptoms

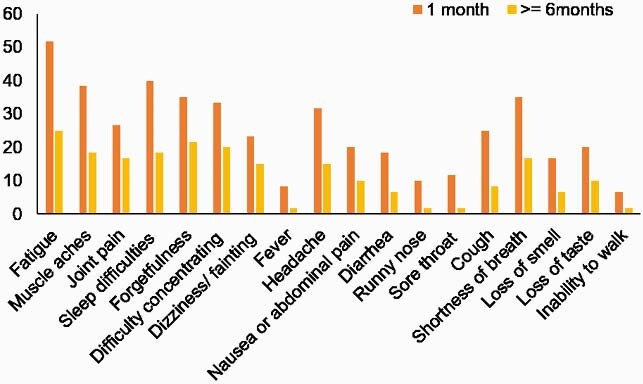

Included if “new since covid”. For 60 subjects consented post-covid with completed questionnaire, results were analyzed. Most common symptoms reported were fatigue/tiredness or exhaustion (52%), muscle aches (38%), difficulty concentrating (33%) and headache (32%) as the most common symptoms during one month prior to their initial follow-up visit. The persistent symptoms experienced for six months or longer were fatigue/tiredness or exhaustion (25%), forgetfulness (22%), muscle aches (18%), and sleep difficulties (18%).

**Results:**

Cohort is 36 (60%) female, 24 (40%) male, age group of 49 (82%) 18-64 years, 11 (18%) 65+ years, 33 (55%) African American, 27 (45%) Caucasian. Median follow-up time after symptom onset: 290 days. Study cohort reported fatigue (52%), myalgias (38%), difficulty concentrating (33%), headache (32%) as most common symptoms during first month from initial symptom onset. Persistent symptoms ( >=6 months) are fatigue (25%), forgetfulness (22%), myalgias (18%), sleep difficulties (18%). Bivariate analysis shows that gender (female, P=0.04), past stroke/transient ischemic attack (P=0.04), deep venous thrombosis (P=0.02), abnormal kidney function (P=0.01) associate with PCS. Convalescent antibodies (ReSARS N IgG, S-RBD IgG) were measured and percentage inhibition of ACE2 spike interaction was recorded. Plasma inflammatory protein levels were measured using multiplex ELISA and Proximity Extension Assay technology during follow-up visit. Increased antibody ReSARS N IgG (2.91, 0.74-10.93; P=0.02) response and higher convalescent IL-10 (P=0.04) was associated with PCS. Percent inhibition of ACE2: spike interaction was not associated (P=0.79) with PCS. Nasal swab/saliva SARS-COV-2 sequencing has not identified a specific SARS-CoV-2 virus mutation predictive of PCS.

Table 1. Demographic and Clinical Characteristics

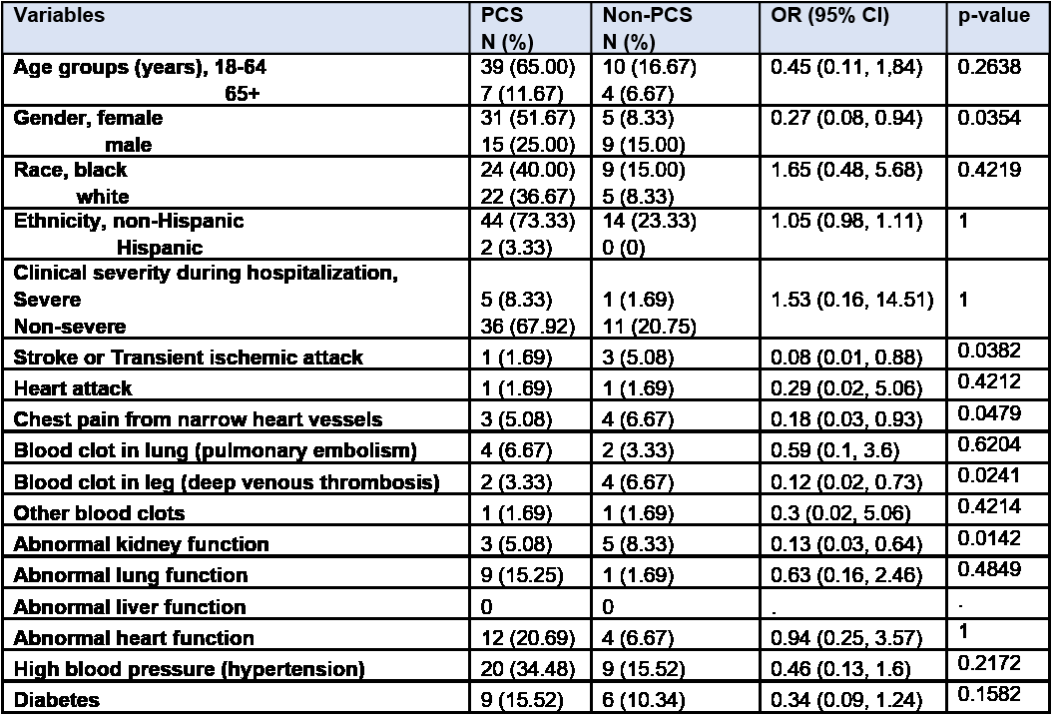

The bivariate analysis results showed that the gender (female, P=0.0354), history of stroke or transient ischemic attack (P=0.0382), chest pain from narrow heart vessels (P=0.0479), deep venous thrombosis (P=0.0241) and abnormal kidney function (P=0.0142) were associated with Post-COVID syndrome.

Table 2. Antibodies and ACE2 spike inhibition.

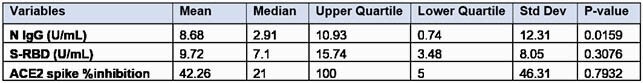

The convalescent antibodies, ReSARS N IgG and S-RBD IgG were measured in U/mL and percentage inhibition of ACE2 spike interaction was recorded during follow-up visit for PCS vs Non-PCS subjects. The increased antibody ReSARS N IgG (2.91, 0.74-10.93; P=0.0159) response was associated with Post-COVID syndrome. Percent inhibition of ACE2: spike interaction was not associated (P=0.7932) with PCS.

Table 3. Plasma inflammatory protein levels.

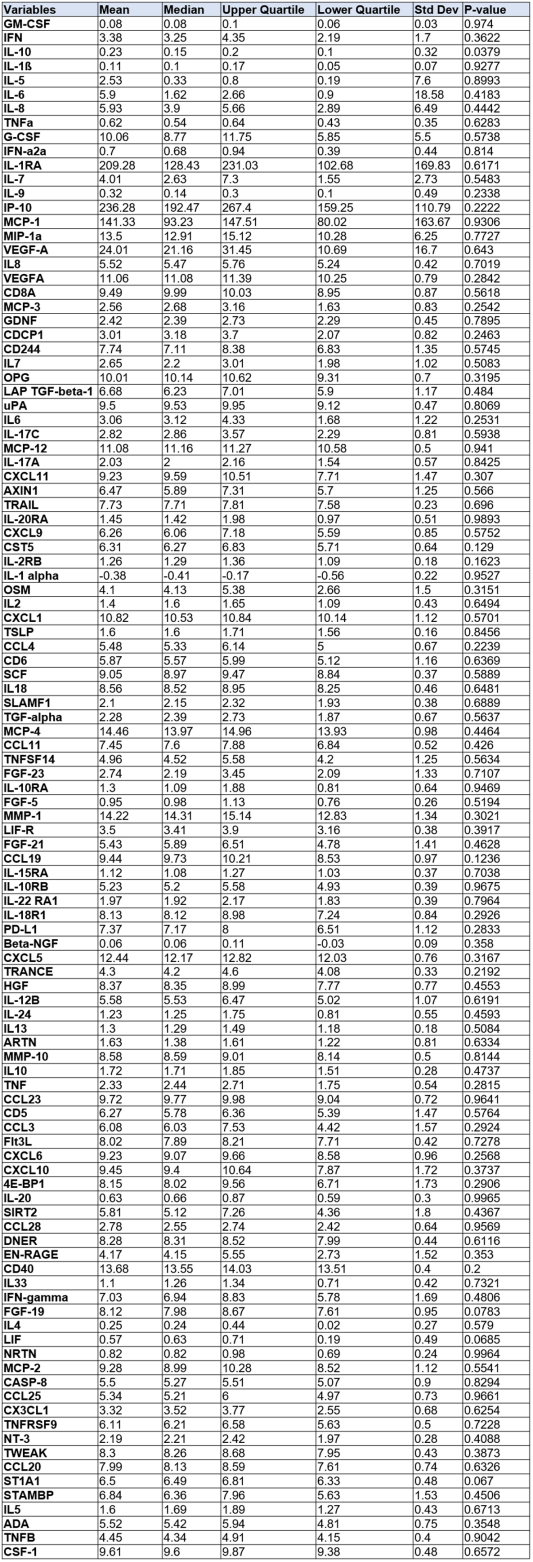

Plasma inflammatory protein levels were measured using multiplex ELISA (MSD) and Proximity Extension Assay technology (Olink) recorded during follow-up visit for PCS vs Non-PCS subjects, revealing IL-10 (P=0.0379) was associated with development of PCS.

**Conclusion:**

This study identifies initial clinical and biomarker predictors of PCS in a cohort that is 55% African American.

Figure 2. Antibody ReSARS N IgG

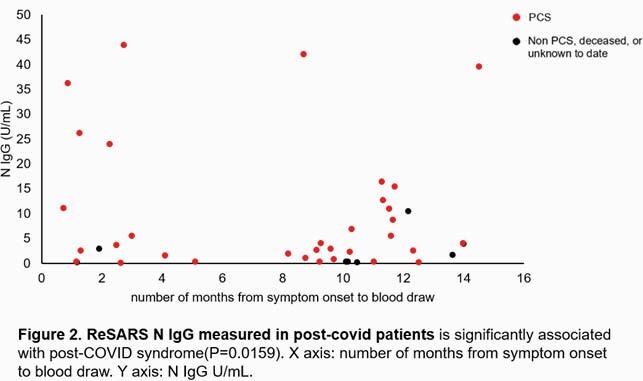

ReSARS N IgG measured in post-covid patients is significantly associated with post-COVID syndrome(P=0.0159). X axis: number of months from symptom onset to blood draw. Y axis: N IgG U/mL.

Figure 3. Spike amino acid mutations

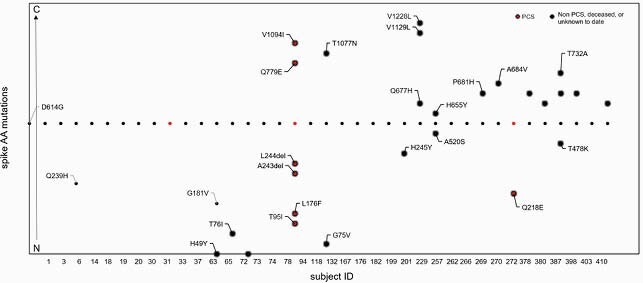

Spike amino acid mutations detected in SARS-CoV-2 from acute-phase respiratory isolates. Nasal swab/saliva samples were collected from subjects with acute COVID-19 at time of enrollment into ClinSeqSer, stored at -80°C followed by RNA isolation and SARS-CoV-2 qRT-PCR. Samples with Ct value of ≤30 were then sequenced using NextSeq (Illumina). All sequences are deposited on GISAID and under BioProject (ID PRJNA681020). X axis: subject ID, with ID number increasing chronologically. Y axis: amino acid position of each mutation moving from N- to C-terminus.

**Disclosures:**

**Robert Garry, PhD**, **Zalgen Labs** (Shareholder)

